# Obesity Hypoventilation Syndrome (OHS) as A Chronic Inflammatory Disease: A Clinical Case

**DOI:** 10.1002/ccr3.72991

**Published:** 2026-06-21

**Authors:** Sajedeh Zarghi, Samira Mawdoodi, Sara Movahed, Mohammad Safarian, Fatemeh Roudi

**Affiliations:** ^1^ Department of Nutrition, Faculty of Medicine Mashhad University of Medical Sciences Mashhad Iran; ^2^ Metabolic Syndrome Research Center Mashhad University of Medical Sciences Mashhad Iran

**Keywords:** malnutrition, obesity, obesity hypoventilation syndrome

## Abstract

Delayed recognition and management of severe obesity and obesity hypoventilation syndrome may result in life‐threatening respiratory failure and multi‐organ complications requiring prolonged intensive care. In metabolically unstable patients, pre‐existing obesity‐related malnutrition may worsen despite obesity itself and contribute to increased mortality. Early multidisciplinary intervention is essential to reduce morbidity and healthcare burden.

**Abbreviations**
AA: Ascorbic Acid (Vitamin C)
ALT: Alanine Aminotransferase
AST: Aspartate Aminotransferase
BID: bis in die (twice daily)
BSA: Body Surface Area
BUN: Blood Urea Nitrogen
CRP: C‐reactive Protein
EN: Enteral Nutrition
HD: Hospital Day
Kcal: Kilocalories
MAC: Mid‐Arm Circumference
NST: Nutrition Support Team
OHS: Obesity Hypoventilation Syndrome
PN: Parenteral Nutrition
PO: Per os (by mouth)
PPN: Partial Parenteral Nutrition

## 1 Introduction

Obesity is a chronic inflammatory disease, relapsing, non‐communicable multisystem disease characterized by an abnormal of excessive body fat that is a most important risk to health [1, 2]. This disease contributes to a reduced life expectancy mainly because of several related complications including cardiovascular diseases including myocardial infarction, type 2 diabetes, hypertension, fatty liver diseases, stroke, mental, obstructive sleep apnea, osteoarthritis, disorders and even some types of cancer disease [3, 4]. Obesity causes are complex and include biological, genetic, environmental, behavioral factors and their interplay [3]. In this context, the brain plays a central role in the pathogenesis of obesity, because it regulates food intake and energy metabolism [3]. Importantly, not every person living with obesity develops typical obesity‐related complications [5]. The heterogeneous association between obesity and its complications despite shared environments maybe explained by genetic and epigenetic factors, but also behavioral patterns that modulate processes and biological pathways involved in the pathogenesis of obesity complications [5]. However, there are cardiometabolic diseases (type 2 diabetes, hypertension, fatty liver diseases, dyslipidemia) that typically occur together and are therefore considered as metabolic syndrome [3]. Also, a common underlying pathology that includes insulin resistance, abdominal fat distribution and chronic inflammation as potential disease primers as the parallel occurrence of these diseases suggests [3, 6, 7]. Inflammation of the Adipose tissue (AT) in obesity is related to a shift of the anti‐inflammatory macrophages in adipocytes from lean individuals to the pro‐inflammatory macrophages and presence of excessive AT enhances lipogenesis and activates the innate immune system [8]. The BMI was significantly increased in the obese group, as was expected, indicating that the obese patients have increased fatty mass and are prone to systemic inflammatory response and local monocyte and neutrophil infiltration in the adipose tissue [9, 10, 11]. The excessive accumulation of fat in the adipose tissue, both by adipocytes' hypertrophy or hyperplasia, causes stress and dysregulation, leading to inflammation, with the low‐grade inflammation turning into chronic and, finally, systemic inflammatory response after a prolonged inflammatory phase [9, 10, 11]. Unfortunately, often these comorbidities do not manifest themselves, and a person with obesity may appear healthy on the outside. Therefore, malnutrition in obesity patient presents as a complex diagnostic challenge due to the distinctive physiological characteristics of this disease. In Western society, the identification of malnutrition has focused on undernutrition due to disease‐related malnutrition and its consequences [12]. While malnutrition is a broad term comprising several forms such as micronutrient deficiencies and protein–energy malnutrition, with conditions like overweight and obesity (hereinafter referred to as ‘obesity’) also being considered as a state of nutritional imbalance [13, 14].

In this study, we intend to investigate a case of class III obesity admitted to the intensive care unit with severe dyspnea. A 48‐year‐old Iranian woman with a past medical history of class III obesity and obesity hypoventilation syndrome (OHS) presented to a community hospital with difficulty breathing, sudden loss of consciousness, and inflammation.

## 2 Case History/Examination

**FIGURE 1:**
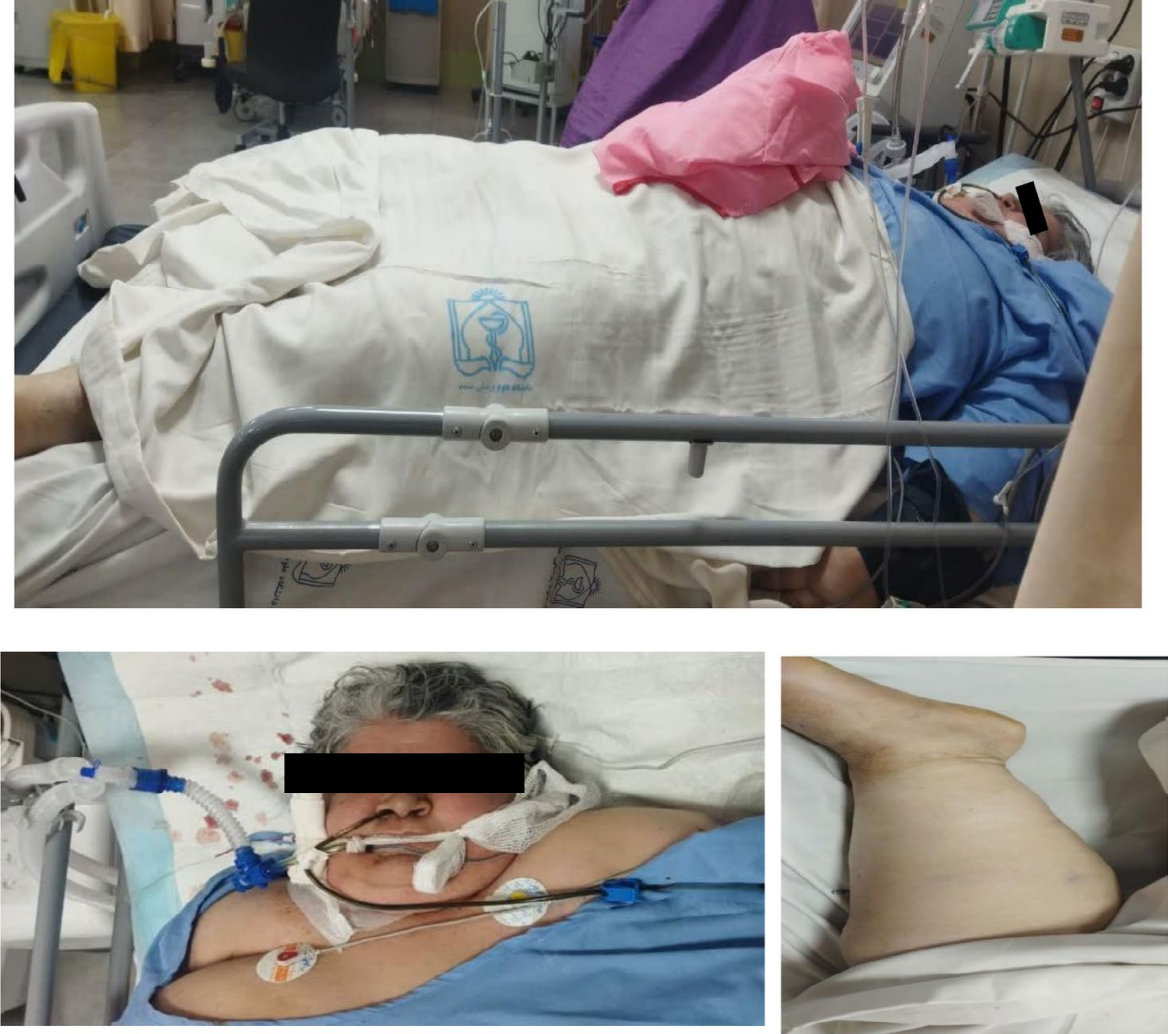
**Case with obesity admitted to the ICU**. Patient's appearance: Height was 160 cm, weight 140.8 kg, and body mass index (BMI) = 55 kg/m^2^.

A 48‐year‐old Iranian woman with a past medical history of class III obesity and obesity hypoventilation syndrome (OHS) presented to a community hospital with difficulty breathing and loss of consciousness. Her height was 160 cm, weight 140.8 kg, body mass index (BMI) 55 kg/m^2^, and mid‐arm circumference (MAC) 60 cm (Figure 1). OHS was defined as the presence of diurnal hypercapnia (arterial PCO_2_ > 45 mmHg) in the context of obesity (BMI ≥ 30 kg/m^2^) and sleep‐disordered breathing [15]. In this patient, PCO_2_ was 46 mmHg, BMI was 55 kg/m^2^, and intubation was required.

On arrival, she was in cardiopulmonary arrest and immediately underwent cardiopulmonary resuscitation (CPR); after 30 min, return of spontaneous circulation (ROSC) was achieved. She was intubated when her arterial blood gas (ABG) revealed hypercarbia. She had no past medical history of previous cardiorespiratory disease or other comorbidities. On suspicion of pneumonia, empiric antibiotic therapy was initiated. Her level of consciousness was estimated as Richmond Agitation‐Sedation Scale (RASS) −4, corresponding approximately to a Glasgow Coma Scale (GCS) score of 5.

According to the results of a brain CT scan, there were evidence of hernia, and sinusitis and cerebral edema. Given the increase in serum sodium from 150 to 155 mL, a vial of 5% sodium was prescribed. Then Evidence of coffee ground secretions was observed in the NG tube and a blood culture for Klebsiella pseudomonas was performed and Klebsiella was evident in the second culture were observed as clinical signs suggesting presence of pneumonia.

Subsequently, oliguria, worsening renal function with rising serum creatinine, and elevated hepatic enzymes developed, which were diagnosed as ischemic acute tubular necrosis (ATN) and ischemic hepatitis, indicative of multi‐organ failure.

She transferred to the medical ICU at our tertiary care center. On arrival, she was hemodynamically stable with normal oxygen saturation on examination; she had profound generalized obesity, a crowded oropharynx, and a large neck. On auscultation, breath sounds were reduced over the right hemithorax, while heart sounds were regular. The abdomen was distended with no palpable organomegaly.

## 3 Differential Diagnosis, Investigations and Treatment

At the time of admission, the Laboratory findings were as follows: creatinine 5.4 mg/dL with a GFR of 9.1 mL/min/1.60 m^2^, hemoglobin 8.8 g/dL, white blood cell counts 17.7 × 10^3^/μL, elevated liver function tests (AST 910 U/L, ALT 1830 U/L), albumin 3.6 g/dL, INR 1.05, and urea 117 mg/dL. ABG showed pH 7.3 and PCO_2_ 54 mmHg (> 45 mmHg). Compared with OSA, OHS has hypoxemia and hypercapnia not only at night but also in daytime. We illustrate ABG in daytimes. ABG criteria (arterial PCO_2_ > 45 mmHg).

Chest radiograph demonstrated findings consistent with right lung collapse and mucus plugging. Cardiac echocardiography revealed a reduced ejection fraction with left ventricular hypertrophy (LVEF 45%). Laboratory tests and nutritional interventions are presented in Tables 1 and 2, respectively. Energy and protein requirements considered approximately 25 kcal per kilogram of adjusted body weight according to ESPEN. Also We used mild arm circumference to estimate the patient's weight and energy, protein, and fluid needs because it is a more accurate measurement than neck circumference based on study Jan MT, et al. but due to sever edema we don't measure it at second weed of hospitalization [16]. Due to the patient's gastrointestinal bleeding, gavage was contraindicated. On the other hand, the unstable metabolic condition prevented the administration of full parenteral nutrition. For this reason, in the first week after hospitalization in ICU, the patient's medical nutrition therapy was performed as a combination of enteral trophic nutrition (50 cc gavage/24 h) and partial parenteral nutrition. For preventing refeeding syndrome, 15 kcal per kilogram of adjusted body weight, 1055 kcal and 93.5 g protein were considered. In the third week, 25 kcal per kilogram of adjusted body weight according to ESPEN was considered. Due to gastrointestinal bleeding, enteral nutrition was completely discontinued and the patient's needs were met only through parenteral nutrition. In the eighth week, enteral nutrition was administered at a rate of 200 cc every 3 h, but the patient still had residue in some meals, and parenteral nutrition was also administered. The patient was ventilated on CPAP (continuous positive airway pressure) mode on the first day after admission to the ICU from the emergency department and did not come off the ventilator. After the second week, the patient underwent tracheostomy after that long hospital stay.

**TABLE 1:** Lab test.

Hospitalization date	Hematology tests			Biochemical tests (mg/dl)											
	WBC	Hb	PLT	Ur	Cr	Alb	Total pr	Na	K	P	Mg	AST	ALT	BS	CRP
Day 1	17.7	8.8	134	117	—	3.6	—	138	5.3	—	2.5	910	1830	285	60
Day 2	—	—	—	146	5.47	3.4	—	140	5.7	4.7	2.18	650	330	—	—
Day 20	18.7	7.9	121	155	2.7	—	—	152	4.1	5.9	—	—	—	—	—
Day 21	19.7	7.9	169	146	2.1	3.6	6.3	155	4.8	5.1	—	61	46	—	14
Day 40	4.2	8.8	20	97	1.6	—	—	144	3.5	4.5	—	—	—	—	137.2
Last day	11.1	7.6	54	105	1.9	—	—	139	4.8	6.0		—	—	—	134

*Note:* Patient laboratory tests during hospitalization.

Abbreviation: BS, Blood Sugar.

**TABLE 2:** Summary of nutrition interventions from the Nutrition Support Team.

Date	Oral	ONS1 ENTRAL\ energy requirement	PPN 2 or serum and supplement	Total intake
June 7, 2025 First week	NPO	Entrameal high protein 50 cc/3 h Preventing refeeding syndrome: 1.2 g protein and 15 kcal per kilogram of adjusted body weight 1055 kcal 93.5 g protein Adjusted body weight ~78.5 (140–58/4) 1‐time residue	Ser D/S 500 cc BID	600 kcal 20 g protein
June 21, 2025 Third week	NPO	NO: GI bleeding 25 kcal per kilogram of adjusted body weight according ~1900 k cal ESPEN	Ser D/S 1000 cc+ + 6vial dextrose 50% + 10 cc KCl Ser Amino Acid 10% 500 cc QOD Ser Intra Lipid 10% 500 cc QOD Syrup Multi vitamin 10 cc Po daily Syrup Zinc 10 cc Po daily Perl vitamin A 50000 IM weekly Perl vitamin D_3_ 50,000 IM weekly Amp apotel 1 g IV daily	1300 kcal 50 g protein
July 5, 2025 Sixth week	NPO	Entrameal high protein 120 cc/3 h 25 kcal per kilogram of adjusted body weight according ESPEN 2‐time residue	Ser D/W 1000 cc+ + 3vial dextrose 50% + 10 cc KCl Ser Amino Acid 10% 500 cc BID Ser Intra Lipid 10% 500 cc QOD Syrup Multi vitamin 10 cc Po daily Syrup Zinc 15 cc Po daily Perl vitamin A 50000 weekly Perl vitamin D3 50,000 weekly Amp solovit IV daily	1800 kcal 100 g protein
July 19, 2025 Eighth week	NPO	Entrameal high protein 200 cc/3 h 25 kcal per kilogram of adjusted body weight according ESPEN 3‐time residue	Ser D/W 1000 cc+ + 4vial dextrose 50% Amp Apotel 1 g IV daily Ser Amino Acid 10% 500 cc BID Syrup Zinc 15 cc Po daily Perl vitamin A 50000 weekly Perl vitamin D_3_ 50,000 weekly Amp vitamin C 500 mg daily Amp B‐complex 1 IV daily	1400 kcal 100 g protein

*Note:* Energy and protein requirement calculation coefficients were calculated based on ESPEN (the European Society for Clinical Nutrition and Metabolism) guidelines with the patient's adjusted weight. At each stage, barriers to achieving nutritional goals, such as active gastrointestinal bleeding or lack of market access to macronutrients and micronutrients, are mentioned.

During her ICU course, she was extubated after correction of hypercarbia but subsequently developed hypoxemic respiratory failure, requiring re‐intubation. Liver enzymes showed a decreasing trend, while renal function remained unchanged. Therefore, routine hemodialysis was initiated. Brain CT revealed cerebral edema and herniation, prompting treatment with a 5% hypertonic saline infusion.

A multidisciplinary team, including sleep specialists, chest physicians, cardiologists, nutritionists, and nephrologists, was involved in the patient's management. Most treatment strategies focused on sleep‐disordered breathing (SDB), with positive airway pressure (PAP) therapy administered during sleep, as each hour of PAP use has been associated with a reduction in PaCO_2_ of approximately 1.8 mmHg. Antibiotics were administered for pneumonia, and hypertonic saline was used for cerebral edema. Routine dialysis was performed three times weekly for nephrological management. A cardiologist was also consulted for cardiac evaluation.

Due to active gastrointestinal bleeding, the patient remained NPO, precluding the prescription of a specific diet. Nevertheless, fluid and nutritional support were adjusted according to the patient's clinical condition and comorbidities. Calorie and protein requirements were estimated based on ICU nutrition guidelines. Because of her prolonged ICU admission and critical condition, other nutritional management strategies, including exercise and bariatric interventions, could not be considered.

## 4 Conclusion and Results

After a 45‐day ICU stay, the patient experienced recurrent cardiopulmonary arrest. Despite 45 min of resuscitative efforts, she ultimately passed away. The poor outcome was likely related to post‐cardiac arrest syndrome, including anoxic brain injury and neurological deterioration, which are major determinants of survival and functional recovery after cardiac arrest. Neurological outcomes in these patients are commonly assessed using the Cerebral Performance Category (CPC) scale, which correlates with functional status, quality of life, and clinical outcome.

## 5 Discussion

Our case represents a complex intersection of morbid obesity (BMI = 55 kg/m^2^) and obesity hypoventilation syndrome (OHS) complicated by cardiopulmonary arrest and subsequent multi‐organ dysfunction. In such scenarios, the disease course can be rapidly progressive and devastating. Previous reports have described malignant forms of OHS leading to multi‐organ failure [17]. In our patient, prolonged cardiopulmonary resuscitation (CPR) resulted in ischemic injury to both the liver and kidneys. Studies have demonstrated that hypoxic hepatitis is a frequent complication after cardiac arrest and is strongly associated with mortality [18, 19]. Similarly, our patient developed marked transaminase elevation and persistent renal failure requiring daily hemodialysis. Obesity, particularly in this case, is a significant risk factor for the development and exacerbation of OHS. The excessive adiposity leads to mechanical and metabolic alterations that compromise respiratory function. Increased fat deposition in the chest wall and abdomen reduces diaphragmatic excursion and chest wall compliance, elevating the work of breathing and contributing to daytime hypercapnia and nocturnal hypoxemia characteristic of OHS [20]. Beyond mechanical effects, obesity induces a state of chronic low‐grade systemic inflammation. Adipose tissue, especially visceral fat, serves as an active endocrine organ secreting pro‐inflammatory cytokines such as TNF‐α, IL‐6, and MCP‐1 [21, 22]. These mediators not only promote insulin resistance but also impair endothelial function, contributing to cardiovascular complications [21]. Obesity leads to chronic activation of immune cells, as excessive nutrient intake and adipose tissue expansion create a pro‐inflammatory microenvironment that stimulates macrophages, T cells, and other immune populations [22, 23]. Adipose tissue macrophages in obese individuals shift toward a pro‐inflammatory M1 phenotype, while T cell populations are skewed toward a pro‐inflammatory profile, including increased Th1 and Th17 activity [22, 23]. These immune changes amplify systemic inflammation and reduce the capacity for effective immune regulation, making obese patients more susceptible to severe inflammatory responses during acute illness [24]. In critically ill obese patients, this baseline pro‐inflammatory state complicates management in the ICU. The persistent systemic inflammation is associated with exaggerated responses to stress, infection, or ischemic insults, contributing to more severe organ dysfunction and prolonged recovery. As a result, patients with morbid obesity are often more hemodynamically unstable, display amplified cytokine responses, and have increased risk of multi‐organ failure compared with non‐obese patients [24]. Airway and ventilatory management in morbidly obese patients are particularly challenging. These patients have higher risks of difficult intubation, atelectasis, and airway obstruction [25]. This aligns with our case, where chest radiography confirmed right lung collapse secondary to mucus plugging.

Neurologically, our patient developed severe cerebral edema and herniation following cardiac arrest, as confirmed by non‐contrast brain CT demonstrating diffuse cerebral swelling. This is consistent with prior reports indicating that massive cerebral edema is common after cardiac arrest and is associated with poor neurological outcome and survival [26]. Despite intensive management by a multidisciplinary team—including daily visits by a nutritionist, close monitoring by ICU specialists, and consultations with pulmonology, cardiology, nephrology, and gastroenterology—the patient did not achieve adequate nutritional intake. Several factors contributed to this outcome. Gastrointestinal bleeding limited the patient's ability to consume or absorb nutrients. In addition, there were limitations in the availability of the prescribed nutritional items within the hospital, which hindered full implementation of the nutritional plan also, due to the high volume of residual gastric contents, the patient could not tolerate the prescribed nutrition, which significantly contributed to inadequate intake. Overall, this case underscores the importance of early recognition and aggressive management of OHS and morbid obesity to mitigate catastrophic outcomes. Tailored ventilatory strategies, vigilant monitoring of organ function, and awareness of potential complications are essential. Nevertheless, as illustrated here, the prognosis of severely obese patients with multi‐organ failure remains poor. In this case, parenteral nutrition was not possible due to multiple metabolic problems. Therefore, we were unable to do anything to improve the patient's condition by providing nutritional support. Since different metabolic disorders accompany the development and progression of obesity [27]. During the progression of obesity from overweight to severe obesity, it may cause different metabolic disorders, which are associated with the alteration of gut microbiota profiles. Therefore, it is critically important to prevent and treat obesity [27]. In addition, the molecular signaling pathways may also change during the progression of each obesity‐associated comorbidity [27]. Strategies for obesity treatment including dietary and exercise modification, bariatric surgery, medicines can be used as obesity management. Finally, a combination of two therapies or multiple treatments from therapies should be applied as soon as possible to prevent hospitalization of obese people.

Among these strategies, bariatric surgery has been shown to be an effective intervention for substantial and sustained weight loss and may improve obesity‐related conditions such as obesity hypoventilation syndrome. However, bariatric procedures are not without limitations. Sleeve gastrectomy and one‐anastomosis gastric bypass may be associated with postoperative complications such as cholelithiasis, gastritis, or gastroesophageal reflux disease, and certain technical factors (e.g., antrum size in sleeve gastrectomy) may influence postoperative outcomes. Therefore, careful patient selection and long‐term follow‐up are essential when considering bariatric surgery as part of obesity management [28, 29, 30].

This case illustrates the severe and potentially fatal consequences of class III obesity in conjunction with obesity hypoventilation syndrome (OHS), conditions that may rapidly lead to cardiopulmonary arrest, multi‐organ failure, and death. The complex interplay of respiratory, cardiac, renal, and hepatic complications renders management of these patients highly challenging. Effective care requires a multidisciplinary approach involving intensivists, cardiologists, pulmonologists, nephrologists, and nutrition specialists. Medical nutrition therapy in critically ill patients like this one is crucial, as individualized nutritional support can significantly influence patient outcomes in the ICU and reduce complications. By reporting this case, we emphasize the importance of early identification and timely intervention in patients with OHS, as well as the need for heightened physician awareness regarding the life‐threatening risks associated with morbid obesity. Moreover, this case highlights that prevention of severe obesity through lifestyle modification, consultation with nutritionists, structured dietary plans, and appropriate physical activity is critical. Public health initiatives should focus on education and accessible programs to prevent obesity before it reaches life‐threatening stages, reducing the burden of OHS and other obesity‐related complications. Such awareness and preventive strategies are essential to improve outcomes and reduce the burden of complications in this high‐risk population. Finally, a combination of two therapies or multiple treatments from therapies should be applied as soon as possible to prevent hospitalization of obese people.

## Author Contributions

**Sajedeh Zarghi:** data curation, investigation, writing – original draft, writing – review and editing. **Samira Mawdoodi:** data curation, investigation, writing – original draft, writing – review and editing. **Sara Movahed:** data curation, investigation. **Mohammad Safarian:** writing – review and editing. **Fatemeh Roudi:** supervision, writing – review and editing.

## Funding

This study was not supported by any specific funding from public, commercial, or not‐for‐profit agencies.

## Ethics Statement

Written informed consent for publication of this case report and accompanying images was obtained from the patient's son (legal guardian).

## Consent

All authors gave their consent to publish this study.

## Conflicts of Interest

The authors declare no conflicts of interest.

## Data Availability

The authors confirm that the data supporting the findings of this study are available within the article.
